# Physical activity promotion in an urban district: Analyzing the mechanisms of interorganizational cooperation

**DOI:** 10.1371/journal.pone.0260053

**Published:** 2021-11-15

**Authors:** Hagen Wäsche, Laura Wolbring, Alexander Woll

**Affiliations:** Institute of Sports and Sports Science, Karlsruhe Institute of Technology, Karlsruhe, Germany; University of Bucharest, ROMANIA

## Abstract

Past research has identified the importance of cooperation among community-based organizations from different sectors to address public health problems such as insufficient physical activity. However, little is known about how and why interorganizational cooperation occurs. The present study sought to analyze the structure and emergent patterns of interorganizational cooperation within a network promoting physical activity based in an urban district neighborhood of a city in Southwestern Germany. Survey data on cooperative relations among 61 network organizations and organizational attributes (e.g., possession of sport facilities) were collected. Social network analysis was applied to examine network properties and exponential random graph models were estimated to test hypotheses concerning mechanisms and conditions of cooperative tie formation. The results show that the network of cooperation is sparse but characterized by a tendency for cooperation to occur in triangular structures. Other significant mechanisms of cooperative tie formation are preferential attachment, with the community department for education and sports being the most central network actor, and heterophily regarding the cooperation of organizations from different sectors. This study provides valid and reliable findings on conditions of network formation and significant mechanisms of interorganizational cooperation in the field of physical activity promotion. Knowledge about these mechanisms can help to manage networks effectively and efficiently and reveal potentials for improvement and intensification of interorganizational cooperation in both the present and other research areas of health promotion.

## Introduction

Insufficient physical activity (PA) is a leading risk factor for global mortality [1]. The results of numerous longitudinal studies show that lack of PA is associated with the development of non-communicable diseases, such as coronary heart disease, diabetes mellitus type 2, dementia, and some mental disorders [2]. Globally, about one in four adults is not active enough with this number being even higher in high-income countries [3]. The World Health Organization [4] recommends several policies to enhance PA, which include among others to create active environments with easily accessible places, opportunities, and programs that support an engagement in regular PA.

In this context, communities and their neighborhoods play a crucial role in providing the physical and social environment for people living there. As Bauman et al. [5] found out, the existence of organized sport structures and recreation facilities in the immediate surroundings is of great significance when it comes to PA participation. This goes hand in hand with the approach that health extends beyond the individual actions of a single person and depends on structural developments and environmental conditions, which also include the organizational level [6]. Thus, physical activity promotion (PAP) is a crucial challenge of sustainable urban development since “a different balance of environmental factors may be required to better support participation in community-oriented sport, recreation and physically active leisure” [7 p373].

Past research in the field of sports, recreation, and health has identified the increasing importance of partnerships, linkages, and cooperation of community-based organizations from different sectors to solve public health problems such as insufficient PA levels that cannot be tackled by one single agency [8–13]. Interorganizational community networks can create synergy effects and reduce duplication efforts by exchanging resources, information, and expertise of involved actors. This, in turn, may improve the efficiency and enhance the capacity of a community to bring different players together to solve challenging community problems and generate greater public awareness [14–17].

Social network analysis (SNA) is a helpful tool to understand which actors are involved in a network, to learn how the network is structured, and to find out which new relations might be highly valuable to develop [10,18]. In addition, it can predict cooperation and effectiveness in organizations as well as potentials for improvement [19,20].

Previous studies have analyzed the structure of these networks but did rarely examine the determinants of network emergence [21–23]. To understand the key aspects, conditions, and causes of cooperative tie formation will help to derive measures on how to develop and manage networks aiming at PAP.

Therefore, the purpose of the present study is to investigate the structure and emergent patterns of cooperation within an interorganizational PAP network based in an urban district of a city in Southern Germany. We aim to examine not only the quality and structure of cooperation but also the types of structural (network-related) and attributive (actor-related) effects that proved to be significant for the formation of interorganizational cooperative ties. Based on this, findings on the development and governance of such networks should be derived.

### Network perspective in public health research

Network research is based on a relational perspective, which means that interesting phenomena are explained by underlying structures. Individuals or organizations are embedded in this structure and do not act in isolation but in mutual dependence. Thus, it is not the individual social actors that are the unit of investigation but their relationships to each other [24–27].

SNA has its origins in the 1930s, when it was first applied in sociology and psychology [28]. Nowadays, network analysis is a largely established research approach that is used in disciplines, such as political science, organizational theory, computer science, mathematics, as well as public health [29,30]. It has been employed in nearly every area of (public) health research, including adolescent risk taking [31], bullying [32], community-based participatory research [33], obesity and PA [34], as well as community coalitions and interorganizational relations [21,35]. Luke and Harris [30] distinguish between three categories of public health networks: Transmission networks, social networks and organizational networks. The latter are seen as one of the most useful public health approaches to share resources and knowledge in order to improve population health [8]. Organizational networks investigate the ties and interactions between agencies or organizations by taking a systems approach [30]. The underlying idea is that public health problems are very complex and multifaceted, however, the public means to solve these problems are generally scarce. Thus, cooperation of public and private organizations from various sectors is important to unite different core competencies and resources in order to develop solutions together in a multisystemic approach. Especially cross-sectoral cooperation beyond the health sector is needed to tackle these problems by joining different perspectives [8,36]. To address public health problems most effectively, it is particularly promising to foster networks on the community level as this is the setting where people live, work, learn, and exercise [12,36–39].

It is assumed that the more ties are realized within interorganizational networks, that is, the more working relationships characterized by trust and mutual support are established and the greater the diversity of available resources, the higher the probability that positive results will be achieved [40].

Based on the structural properties and configurations of interorganizational networks, conclusions can be drawn for network governance, which is essential to manage a network effectively. Three different forms of networks governance can be distinguished [41], which also apply to the field of sports and PA [42]: Firstly, there are participant-governed networks, which represent a highly decentralized form where the network is completely governed by the organizations comprising it. The second type are lead organization-governed networks, describing highly centralized networks which are governed by a single network member. Finally, there are network administration organization-governed (NAO) networks, which also represent a centralized form, however, the leading role is taken by an external organization that is not part of the network. The effectiveness of the different types of network governance is determined by four predictors: distribution of trust throughout the network (density), number of network participants (size), network goal consensus, and the need for network-level competencies such as coordinating and task-specific skills.

### Interorganizational networks to promote physical activity

Several studies have examined interorganizational PAP networks revealing mixed results concerning network properties and structure [43]. This can be attributed to the fact that types of network organizations varied significantly, as did the administrative levels (community, regional, national) at which they operated. In addition, previous studies differ both in terms of the types of cooperation considered and the degree of network formalization, i.e. formally established vs. organically grown networks.

While there are some studies that examine interorganizational PAP networks descriptively [9,19,35,44–47], there are only few studies using statistical modeling and explanatory network analysis to identify relevant patterns of network emergence [21–23]. Results concerning cooperative tie formation are also heterogeneous and strongly depend on the types of organizations involved, the aim of the network, and the conditions of the specific setting and environment, highlighting the need for further analysis.

Based on the idea that the relationships between community-based organizations offering and promoting PA are of decisive importance for the design of urban space and the availability of PA programs, the following study uses SNA to capture, visualize, and evaluate how interorganizational cooperation is structured in a local network promoting PA. Moreover, it aims to reveal underlying mechanisms and conditions of cooperation. Consequently, not only network properties were examined but also several hypotheses concerning the emergence of cooperative ties between the network organizations were tested.

The hypotheses include both endogenous (structural) network effects based on frequently detected configurations of cooperation in self-organizing networks [48] and exogenous (attributive) effects related to organizational characteristics which might also predict tie formation. The following hypotheses were derived.

Centralization is an effect that can often be observed in networks [49,50]. It occurs when network ties are unequally distributed so that a few actors have more ties than others. This results in a preferential attachment effect, where these few actors take a powerful role within the network and have a great influence on network processes. As a result, more and more actors tend to form a connection to the popular actors making them even more powerful. As this effect is frequently observed in interorganizational networks, the relevance of preferential attachment in PAP networks was of interest. Therefore, this study investigated if PAP organizations tend to form cooperative relationships to popular organizations.

Hypothesis 1: PAP organizations form more cooperative ties to popular organizations.

Another phenomenon often observed in networks is the closure of triangles representing an effect of network closure [51]. This effect occurs when a path from actor A to actor B to actor C is closed by a tie from actor C to actor A. The closure of triangles can be seen as an expression of the propensity of actors to act in group-like patterns based on reciprocal support and social trust, which is a significant characteristic of interorganizational networks [52]. The closure from A, B, and C to a closed triangle is an indication that a cooperative relation from C to A (or vice versa) has emerged whose reliability has been approved by a shared neighbor, namely B. This effect is also known as transitivity. It was hypothesized that PAP organizations were more likely to form triplets of cooperation.

Hypothesis 2: PAP organizations form triplets of cooperation.

Homophily refers to the principle that social actors tend to form ties to actors that are similar to them rather than to those that are not similar to them. However, in the present network, the opposite mechanism of working across sector boundaries in multisectoral clusters could play a more important role concerning the formation of ties, as advocated by previous studies [22,53]. A possible explanation for this is provided by resource-dependence theory, which assumes that organizations form heterophil ties to other actors to get access to more diverse information or resources than that available through homogenous ties [54]. Consequently, this also allows for capacity building and the elimination of structural holes as resources are made accessible to others [17,55]. Therefore, it was hypothesized that PAP organizations of a dissimilar type (from different sectors) will develop more cooperative ties among each other, indicating a heterophily effect.

Hypothesis 3: PAP organizations from different sectors develop more cooperative ties among each other.

Not only heterophily might lead to cooperative ties between organizations but also a higher cooperation activity of organizations based on their specific attributes could play an important role. Some PAP organizations might have their own facilities to carry out their sports activities while others do not. Organizations that own a sports facility could therefore show a higher activity in creating cooperative ties as other organizations that do not have a sports facility are dependent on them. Thus, we tested whether the possession of sports facilities results in more cooperative ties.

Hypothesis 4: PAP organizations which own a sport facility show a higher activity in developing cooperative ties.

## Methods

### Sampling and procedure

The current study was carried out in the context of an urban real-world laboratory [56,57]. The setting was a district of the city of Karlsruhe in Southwestern Germany. The district has about 22,000 inhabitants, 42.2% of whom are female and 57.8% male. It is considered as a mature, typically European district that can serve as a model for other urban living spaces in Europe. To locate eligible participants of the network, a systematic search for sports and PA offerings was carried out. Organizations were included if they either owned a sports facility or provided sports and PA programs in the corresponding district. Based on a broad concept of sports, not only traditional and commercial sports facilities and providers, such as sports clubs and fitness centers, but also institutions offering sports and PA programs, such as schools and old people’s homes, were included. In addition, organizations which assumed superordinate, administrative and advisory functions concerning sports and PA in the city district were taken into account. In this particular case, the location of the latter organizations did not necessarily have to be in the city district of interest.

72 potentially relevant organizations were identified and invited to participate in the study. Data were collected through a web-based questionnaire which was sent to the organizations via e-mail. Different questionnaires were created for each of the following organizational types: Sports clubs, schools, kindergartens/daycare, sports administration and other sports providers (e.g., private sports providers, religious institutions, care facilities).

The study was conducted in accordance with the Declaration of Helsinki and was approved by the institutional review board of the Institute of Sports and Sports Science, Karlsruhe, Germany. All participants gave their written informed consent before participation. 39 organizations (54.2%) participated in the survey and provided usable data. 33 organizations (45.8%) did not participate despite multiple reminders (via e-mail and phone) (41.7%) or due to incorrect or missing information (4.2%). The percentages in parentheses all refer to the 72 identified organizations that were invited to participate in the survey. If an organization had indicated a cooperation with other organizations that had not taken part in the survey, this relationship was symmetrized. Since binary data distinguishing only whether a relationship exists or not and cooperative relations are inherently reciprocal, any cooperative tie from one institution to another could always be regarded as undirected and symmetrical [58]. Through symmetrization, relationships of a total of 22 organizations that did not participate in the survey themselves could be reconstructed, which resulted in a network consisting of 61 organizations (84,7% of invited network actors).

### Measures

Organizations were asked whether they possessed a sports facility in the district of interest. If so, they were supposed to indicate the location, type and other characteristics of the sports facility.

To survey the cooperative ties between the organizations, participants received a list of all identified organizations and were asked with whom they cooperate concerning sports and PA offerings in the corresponding city district. They could name up to ten cooperation partners and had to indicate at least one of the four following types of cooperation for each partner: exchange of information, exchange of personnel, cooperation in the provision of sports and PA programs, and use of sports facilities. Please also refer to S1 Survey items for detailed information on the questionnaire used for data collection.

### Data analysis

For further analysis, the organizations were assigned to three different sectors: the public sector (e.g., schools, universities, health insurances, community departments, public kindergartens), the private sector (e.g., for-profit sports providers and practices), and the non-profit sector (e.g., sports clubs). As in previous studies [21], the different types of cooperative ties were joined into one matric and the network was dichotomized, where 0 indicated no tie and 1 indicated the existence of any type of cooperation (exchange of information, exchange of personnel, cooperation in the provision of sports and PA programs, cooperation in use of sports facilities). Thus, when cooperation is referred to in the following, all types of cooperative ties are considered together.

Descriptive network properties were analyzed with the software package Ucinet (version 6.721) [59] and corresponding network visualizations were created with the software package Visone (version 2.19) [60]. On the node level, degree centrality (CD), betweenness centrality (CB), and eigenvector centrality (CE) scores were examined. While CD refers to the actors’ number of direct ties to other actors, CB can provide insights into how often an organization lies on the shortest path between two other organizations. The higher the CB of an actor, the more control he has over the communication and flow of information within the network. Furthermore, CB can identify actors who could assume a coordinating role concerning network processes [61]. CE measures the importance of an actor by also taking into account the centrality of the nodes the actor is connected to. On the network level, average degree (average number of cooperative ties), density (ratio of realized ties to maximum possible number of ties), the global clustering coefficient (number of closed triangles divided by the total number of closed and open triangles), average distance (average shortest path between a set of two organizations), and degree centralization (extent to which all ties of the network are organized around a few central organizations) were analyzed.

To test the hypotheses concerning mechanisms and conditions of cooperative tie formation, exponential random graph models (ERGMs) were estimated. They offer a suitable solution to analyze how and why social networks emerge as these models allow predictions about the likelihood and rules for the occurrence of cooperative ties between actors based on organization and network properties [62,63]. ERGMs take into account the interdependence of observations, i.e. that one relationship within the network also influences the other relationships in the same network [48]. It is assumed that social networks are composed of smaller micro-configurations, such as triangles or stars, through which the network pattern can be described. Supposed that social networks are subject to principles of self-organization and interdependence of tie formation, ERGMs allow inferences about whether specific micro-configurations are more frequently observed in the network than might be expected by chance. This can then be used to identify social processes that could lead to these structural characteristics. Besides structural micro-configurations, called endogenous network effects, ERGMs can also be used to analyze exogenous network effects, that is, specific attributes of the actors and their influence on tie formation. The results indicate which of the configurations occur more often or less often (positive or negative value for each parameter) than expected based on the existing conditions [62].

Mathematically, “this approach models the probability that a relation exists […] as a linear function of predictors” [64 p105]:

$$P(X)=\frac{1}{\kappa (\theta )}exp\left(\sum_{i}\theta_{i}s_{i}(X)\right)$$

ERGMs explain the global pattern of an observed network, represented by *X* in the formula, as a function of statistical parameters *θ*_*i*_ and micro-structures *s*_*i*_*(X)*. The probability of the investigated network *X* is expressed as a function of the local configurations *s*_*i*_*(X)*. Since this involves a probability distribution, the formula contains the normalizing quantity *κ(θ)* so that the probability of the investigated network ranges from 0 to 1. Similar to regression, *X* represents the dependent variable, the local configurations *s*_*i*_*(X)* represent the predictor variables, and the respective parameters *θ*_*i*_ indicate how important *s*_*i*_*(X)* is in determining *P(X)*. The micro-structures or predictor variables *s*_*i*_*(X)* can both represent endogenous or exogenous network effects. As there are many distinctive local configurations *s*_*i*_*(X)* that can determine the structure of *X*, researchers make a selection based on the hypotheses they wish to investigate. The statistical parameter *θ*_*i*_ allows, by simultaneously considering other effects in the model, inferences about whether the specific micro-configurations *s*_*i*_*(X)* are more frequently observed in *X* than might be expected by chance. So, if we observe a higher quantity of local micros-configurations *s*_*i*_*(X)* in *X* than would be expected when the ties were randomly formed, we have evidence of the prominence of *s*_*i*_*(X)* to account for the global structure of the network *X*. Therefore, if a parameter value associated to *s*_*i*_*(X)* is positive (negative), we can assume that these configurations can be observed more often (less often) in the network than would be expected by chance, which provides evidence for (against) the process associated with such configurations [62].

In other words, the existence of a relation within a network can be predicted from different variables, which represent specific configurations the tie is involved in. The positive or negative value of an estimated parameter indicates the significance of this specific configuration for the emergence of a tie.

**Fig 1:**
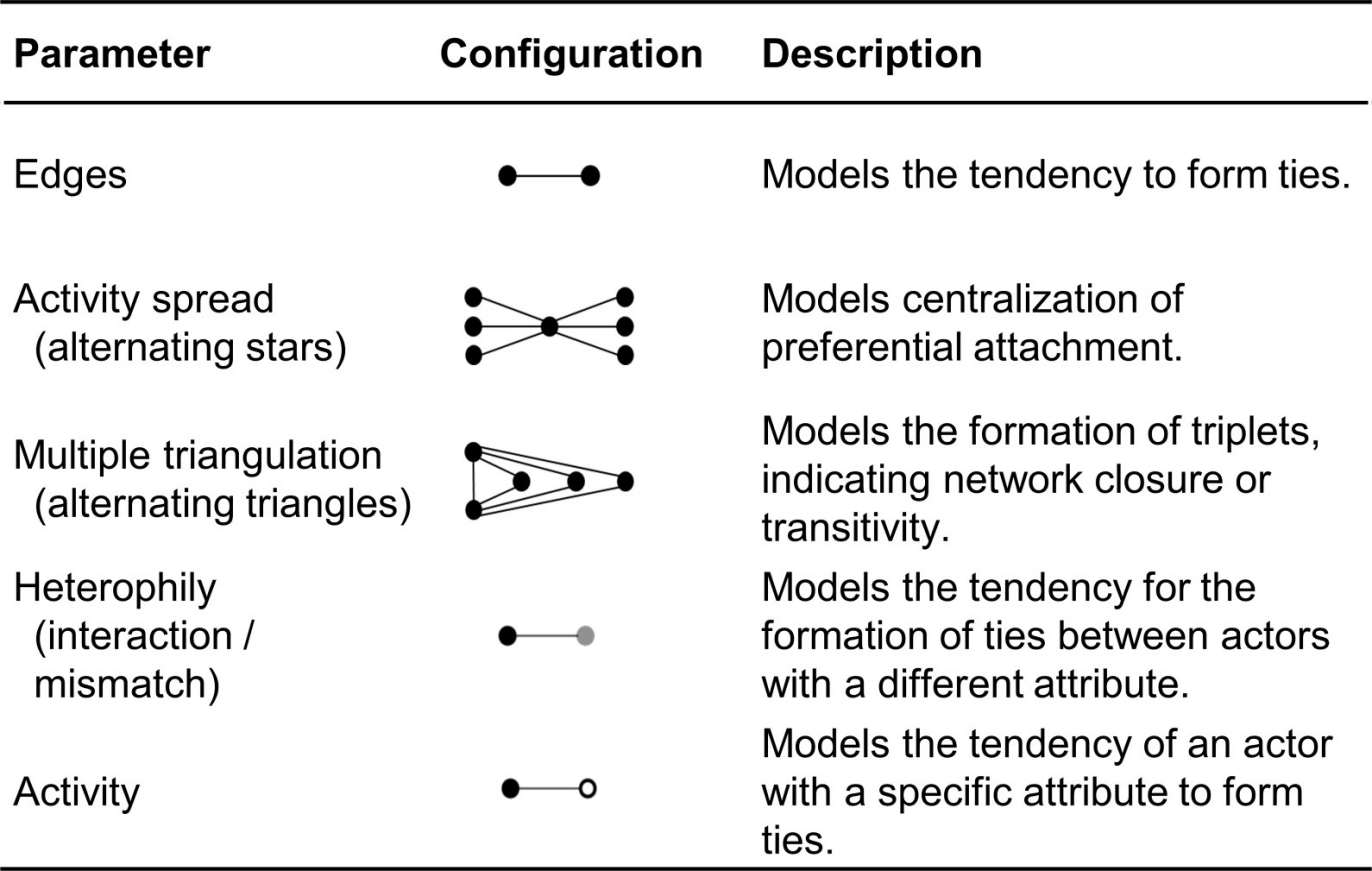
Description of included ERGM parameters.

Markov chain Monte Carlo maximum likelihood methods were used to estimate the parameters for each configuration. Two models were estimated: The first model included only structural (endogenous) network effects. In reference to the previously established hypotheses, alternating stars, indicating network centralization, and alternating triangles, indicating network closure, were estimated as structural parameters. In the second model (full model), attribute-related (exogenous) network effects were added. The following two attribute parameters were included: Mismatch (heterophily effect) refers to the cooperative ties between the three organizational sectors and activity refers to the hypothesized higher cooperative activity of organizations that possess a sports facility. Included endogenous and exogenous parameters and their specific graph configurations are displayed in Fig 1. ERGMs have been estimated with the software package Pnet (version 1.0) [65].

## Results

### Descriptive analysis

**Fig 2:**
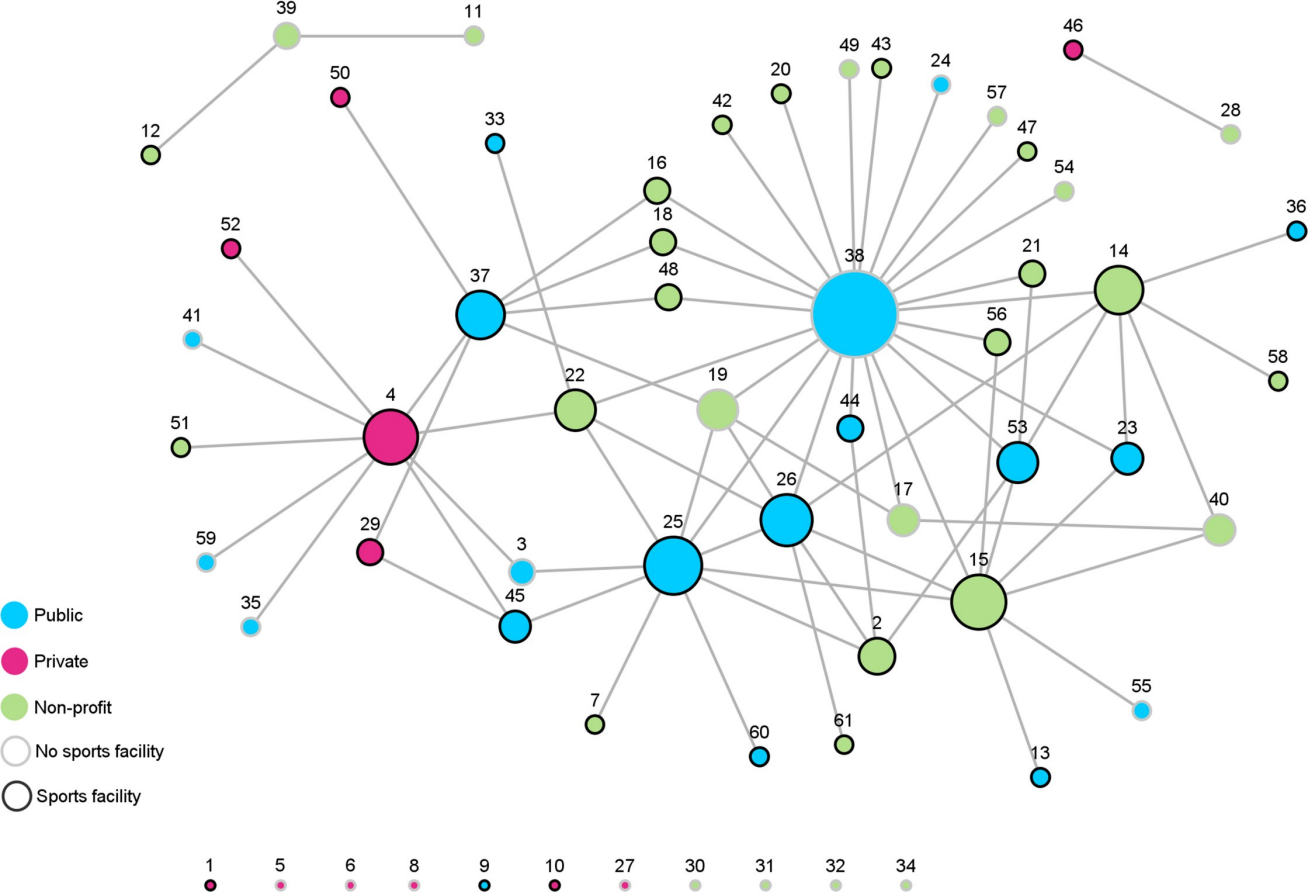
Visualization of the PAP network of cooperation (*n* = 61). Ties between nodes indicate cooperation, node color represents sector affiliation, node boarder color represents possession of sports facility, node size represents degree centrality score (number of collaborative ties to other organizations).

The analyzed network consisted of 61 actors (see Table 1 for the complete list of actors). Most of the organizations were non-profit oriented (50.8%), 31.2% belonged to the public sector and 18% to the private sector. 60.7% of the organizations owned a sports facility, correspondingly 39.3% did not. 50 of the 61 (82.0%) actors had realized cooperative ties, whereas eleven organizations were isolated, most of which were organizations from the private sector or kindergartens (see Fig 2). Overall, there were 74 edges in the analyzed network. Since cooperation is undirected, the network consisted of 148 ties, resulting in a density of 0.04. Thus, only 4% of possible ties had been realized. The average degree was 2.4 (SD = 3.6). Both density and average degree indicate that the network is relatively sparse. The global clustering coefficient was 0.21, pointing towards some tendency that cooperation in the PAP network occurs in triangular structures. The average distance was 2.7, which means that if an organization wants to communicate with another organization with which it is not directly connected, on average, almost two organizations have to act as bridging agents.

**Table 1:** List of network actors.

Id	Name	Id	Name
1	Private fitness center	32	Non-profit kindergarten IV
2	Provider of educational sports and exercise programs	33	Public kindergarten
3	Health insurance I	34	Non-profit kindergarten V
4	Private health center	35	Health insurance II
5	Yoga school	36	Community department for horticulture
6	Personal training	37	University institute for sports I
7	Cultural institution for children and young people	38	Community department for education and sports
8	Physiotherapy practice I	39	Union of local sports clubs
9	Public after-school care center	40	Association of local sports clubs
10	Tai Chi and Qigong school	41	Health insurance III
11	Religious institution I	42	Local sports club VI
12	Religious institution II	43	Local soccer club II
13	Public old people’s home	44	Local sports club VII
14	Educational outdoor park	45	University institute for sports II
15	Local sports club I	46	Administration of local swimming centers
16	Local sports club II	47	Local sports club VIII
17	Local sports club III	48	Local sports club IX
18	Local soccer club I	49	Scout tribe
19	Local sports club IV	50	Physiotherapy practice II
20	Dancing club	51	Local sports club X
21	Tennis club	52	Midwife practice
22	Local sports club V	53	Public school V
23	Public school I	54	Karate school
24	Public school II	55	Community social and youth authority
25	Public school III	56	Local soccer club III
26	Public school IV	57	Local sports club XI
27	Private kindergarten I	58	City youth committee
28	Non-profit kindergarten I	59	Health insurance IV
29	Private kindergarten II	60	Provider of educational outdoor programs
30	Non-profit kindergarten II	61	Provider of educational circus programs
31	Non-profit kindergarten III		

**Fig 3:**
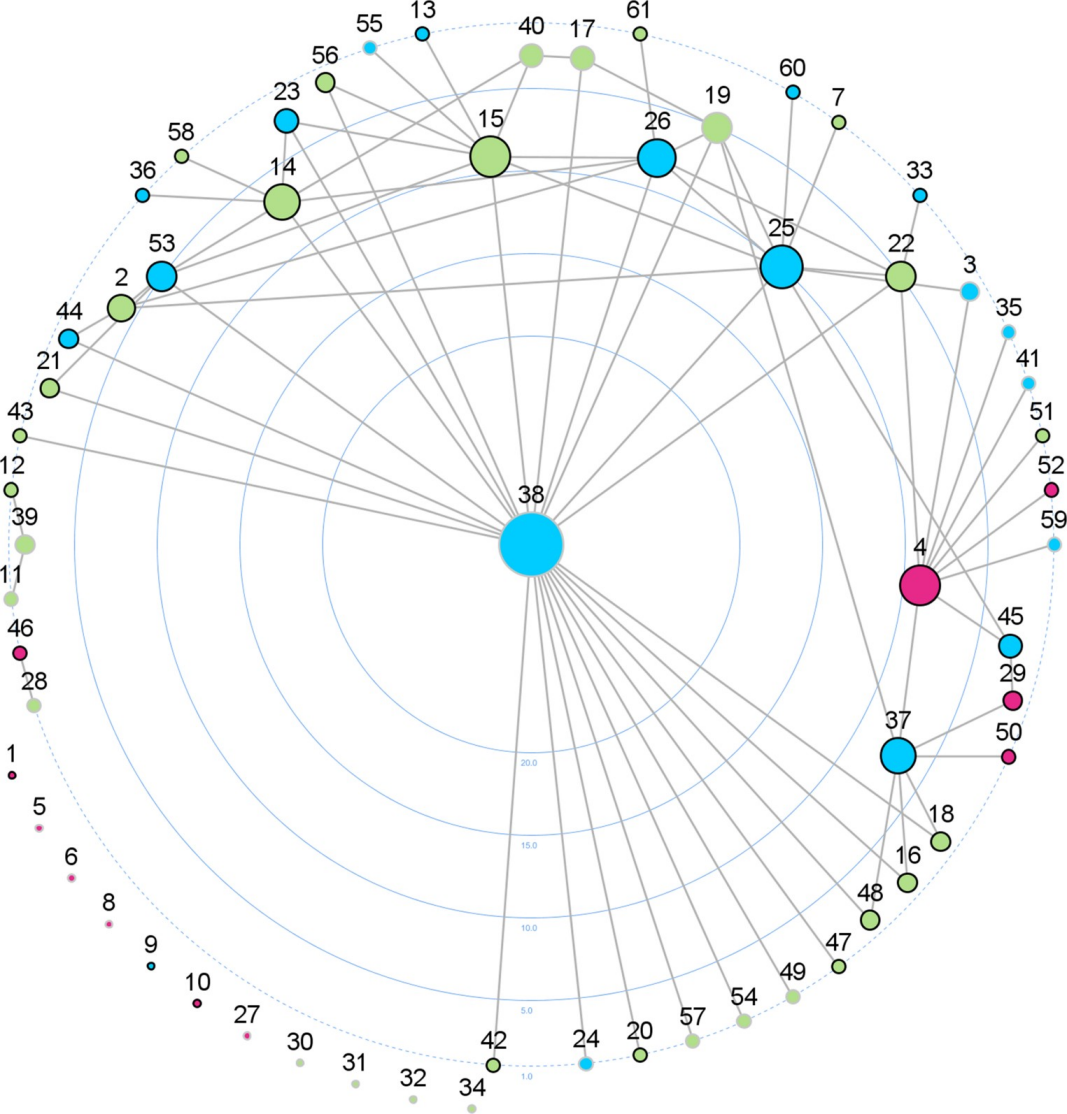
Degree centrality visualization (number of ties) of the PAP network of cooperation (*n* = 61).

The number of ties as well as the normalized centrality scores for degree, betweenness, and eigenvector of the 15 highest scoring actors are listed in Table 2. Fig 3 shows the different positions held by the organizations concerning the number of ties, visualized using a centrality layout.

**Table 2:** Number of ties and normalized degree, betweenness, and eigenvector centrality scores of the 15 highest scoring organizations.

Id	No. of ties	Degree	Betweenness	Eigenvector
38	23	0.38	0.31	0.76
25	10	0.17	0.10	0.43
4	9	0.15	0.12	0.11
15	9	0.15	0.06	0.42
26	8	0.13	0.05	0.44
14	7	0.12	0.05	0.31
37	7	0.12	0.06	0.14
19	5	0.08	0.02	0.31
22	5	0.08	0.10	0.28
53	5	0.08	0.01	0.29
2	4	0.07	0.00	0.21
17	3	0.05	0.00	0.19
23	3	0.05	0.00	0.24
40	3	0.05	0.00	0.15
45	3	0.05	0.02	0.09

With regard to CD, the community department for education and sports (actor 38) had the highest number of ties to other organizations and therefore represents the most central actor with respect to popularity followed by the public school III (actor 25), the local sports club I (actor 15), a private health center (actor 4), and the public school IV (actor 26). The large difference in CD between the most central and the second most central actor illustrates the important position that the community department for education and sports occupies. This is also evident in the network visualization (Fig 2). The degree centralization in relation to the whole network is 0.36, illustrating the difference between the CD of the community department for education and sports and all other actors of the network.

The ten highest scoring organizations concerning CB are nearly the same as for CD, only the ranking order is different. The organization with the highest CB is again the community department for education and sports (actor 38), but the private health center (actor 4) moved from the third (CD) to the second position. The third most central position concerning CB is held by the local sports club V (actor 22), followed by the public school III (actor 25), which moved from second (CD) to the fourth position. The local sports club I (actor 15) is the fifth most central actor regarding CB, holding the fourth position in the CD ranking. Only 22 of all organizations held a CB position, while 39 organizations had a CB score of 0 und thus had no influence on communication processes or flow of information.

Regarding CE, almost the same organizations are among the most central but the order is again slightly different. The community department for education and sports is still the most central actor concerning CE. However, it is noticeable that the public school IV (actor 26), which ranks fifth in CD and eighth in CB, is the organization with the second highest CE.

### Exponential random graph models

The results of the two ERGMs are displayed in Table 3. In model 1, two of the three parameter estimates were significant. The edges parameter was negative suggesting that fewer cooperative ties are realized in the network than would be expected by chance, which points to a relatively sparse network. The positive estimate for centralization provided evidence for a preferential attachment effect implying a tendency for cooperation to revolve around a few central actors (hypothesis 1). The parameter for multiple triangulation was not significant in the first model (hypothesis 2). When adding the exogenous network effects (model 2), the negative edges and positive centralization parameter were still significant. Moreover, the second model provided evidence for multiple triangulation (hypothesis 2). Concerning the attribute-related effects, a positive heterophily effect for organizations from different sectors developing more cooperative ties among each other was found (hypothesis 3). The activity effect for organizations that own a sports facility was not significant (hypothesis 4). In summary, hypothesis 1 can be confirmed as a preferential attachment effect could be observed. Hypothesis 2 can also be confirmed, since a multiple triangulation effect was found in model 2. Organizations from different sectors seem to cooperate more frequently, thus hypothesis 3 can be confirmed as well. However, hypothesis 4 must be rejected since organizations that own a sports facility did not show a higher cooperative activity.

**Table 3:** ERGM parameter estimates for the PAP network of cooperation.

	Model 1		Model 2	
Parameter	Estimate	*SE*	Estimate	*SE*
Cooperative ties (edges)	-5.34*	0.29	-5.90*	0.42
***Structural predictors***				
Centralization (preferential attachment)	0.71*	0.13	0.72*	0.14
Multiple triangulation (closure)	0.28	0.15	0.27*	0.13
***Attribute predictors***				
“Sector” heterophily			0.15*	0.13
“Sports facility” activity			0.55	0.26

*SE* = standard error

*p < 0.05.

Goodness-of-fit-statistics showed satisfactory model fit for the final models.

## Discussion

The results reveal specific properties and structures of the analyzed PAP network. First of all, the community department for education and sports held as by far the most central position within the network. This may be due to the fact that it is responsible for the community sports promotion and, e.g., allocates financial resources to sports clubs or coordinates cooperation between sports clubs and schools. The density and the average degree of the entire network were relatively low with a total of eleven isolated organizations, indicating a rather sparse network. Most of the isolated organizations were from the private sector. One possible explanation for this would be that, on the one hand, these organizations might not have a great need for cooperation because they are not dependent on the (financial) resources from the community, as do sports clubs for example. On the other hand, since establishing and maintaining cooperation is costly, profit-oriented organizations might not develop new relationships for social reasons but only if they promise an economic benefit. Among the isolated actors were also some kindergartens. To introduce children to PA at an early age, it is important to develop strategies on how to integrate these institutions into the network so that they can benefit from exchanging information and resources with other organizations.

One question that arises is whether a higher network density would lead to better network results since many ties can be redundant and time consuming and a lower number of ties may be more efficient. Varda and Retrum [66] analyzed several public health collaboratives with the aim of determining specific factors that lead to a successful collaborative. Even though it seems to be quite challenging to define a specific set of aspects, they found out that organizational characteristics and interorganizational mechanisms do appear to affect outcomes. Since it can generally be assumed that public funding in these types of networks is rather limited, a higher density of interactions, growing levels of trust and the availability of a greater diversity of resources can bring added value to the network and increase the likelihood of positive outcomes [40]. In particular, with regard to the promotion of PA, previous research found out that the integration of isolated actors could increase the capacity of a network to promote active lifestyles [9]. Thus, it can be assumed that developing new cooperative ties within the current network is more of an advantage than a disadvantage.

The density of a network also has implications for its most effective governance. While networks with a high density can be controlled by the network members themselves, it makes sense for fragmented networks with a low density to be managed by an external organization [41]. In any case, an important aspect is to define and communicate specific network goals. With these in mind, it is constantly possible to check whether the current processes contribute to the defined goals and what must additionally be initiated in order to achieve them [67].

The results of the ERGMs revealed underlying mechanisms for the formation of cooperative relationships explained by both structural and attributive effects. Regarding the structural effects, preferential attachment could be observed in the PAP network indicating a substantial tendency of organizations to cooperate with organizations that are already involved in a higher number of ties. This effect can primarily be attributed to the community department for education and sports, underlining its powerful position and influence on network processes. Multiple triangulation was also present in the analyzed network, indicating network closure. Therefore, cooperation seems to take place, at least in part, in smaller clusters based on mutual trust and initiated by the organizations themselves as a bottom-up movement. Comparable patterns were also observed in another community network that evolved around sports and physical activity [68].

As far as attributive effects are concerned, ERGMs revealed a heterophily effect indicating that being situated in different sectors appears to be a strong predictor for cooperation in the analyzed PAP network. The mechanism of cooperation occurring in multisectoral clusters was also found in a previous study [22]. The establishment of heterophil relations to other organizations to get access to more diverse information or resources than that available in one’s own sector meets the demand of Bevc et al. [53] to work across boundaries and unite different sectors in public health collaboratives. Hypothesis 4, which presumed that PAP organizations that own a sports facility show a higher activity in developing cooperative ties could not be confirmed. One possible explanation would be that the allocation of public sports facilities is coordinated by an external organization, which is why actors within the network may not need to establish cooperative ties among themselves in order to be able to access sports facilities. Moreover, the majority of the network organizations already owned a sports facility. A reason for this finding is probably that the city where the study takes place supports sports clubs which possess their own sports facilities in favor of providing sports facilities for the clubs. Therefore, especially many sports clubs had no need to cooperate in this regard. In addition, actors who do not possess a sports facility may also cooperate with organizations outside the network boundaries that were not considered in this study.

Since the analyzed network is an informal network and not a formally established one, an appropriate governance form still needs to be developed to manage the network effectively. Referring back to Provan and Kenis’ network governance criteria [41], the overall network density was relatively low with a high degree of centralization, a moderate number of actors and a probably rather low consensus on the goals to be achieved. Accordingly, a lead organization taking over the governance of the network seems to be suitable. Although the community department for education and sports currently occupies a very central position within the network, it is mainly responsible for allocating financial resources and sports facilities and does not act strategically in the sense of a leadership role. In this respect, it remains to be determined whether the community department for education and sports should assume this role in the future or whether another organization would be more suitable. The role of the lead organization can also be assumed by several central actors, who could then form a leading group to manage the network [42]. The properties of the analyzed network further suggest that cooperation often takes place in small triangular clusters characterized by mutual trust. These clusters consist of only a small number of participants who are in close and reciprocal contact and pursue common goals. For these small networks within the large network, a shared governance form might be appropriate, in which the participants themselves take over the governance [41,42]. Therefore, a hybrid of a lead organization- or leading group-governed network and a participant-governed network might be most effective to manage and develop the analyzed PAP network.

The current study has some limitations that should be considered when evaluating the results. Despite repeated reminders, some organizations were unwilling to take part in the survey, so that probably not all cooperative relationships could be assessed. Therefore, we reconstructed as many of the ties as possible by symmetrization. In addition, the organizations surveyed did not have a uniform, precise understanding of the boundaries of the city district whereupon the radius was extended by 500 m, so that organizations near the boundary of the district were also included. Another limitation could be that the organizations’ contact persons who answered the questionnaire did not know in detail about all cooperative relationships. It should also be noted that a network analysis can only provide a snapshot of the cooperative activities existing at the time of the survey. Moreover, the survey referred to a specific city district which is part of a larger network of the whole city. Consequently, the results cannot be generalized without further elaboration. However, studies of specific networks like this are still the most common approach in network research as they are able to provide insights into the phenomena and mechanisms of a rather new research field. To add to a better understanding of interorganizational networks providing sports and physical activity, future studies should compare and summarize results of similar networks.

## Conclusions

The current study reveals specific characteristics of the interorganizational PAP network and enables an understanding of how cooperation in this network works. Descriptive results such as the identification of isolated and central actors can provide starting points for which central actors should be used to disseminate information and how isolated or peripheral actors can be integrated in order to increase network interaction, cohesion and trust. Furthermore, the ERGMs show valid and robust findings on conditions of network formation and significant mechanisms of interorganizational cooperation, such as preferential attachment, closure or sector heterophily. This can provide valuable knowledge for developing measures on how to intensify existing and establish new cooperative ties among organizations and how to manage networks effectively and efficiently with the aim of promoting PA in an urban setting. For the present network, a first step would be to bring the organizations together and identify and define common goals that everyone is working towards [67].

Based on the similarities of the study findings with other network studies [22,68–70], future research should begin to establish a theoretical framework by which recommendations for network development can be derived. SNA and especially stochastic network analysis are relatively new approaches in PA and sports sciences [27]. Future studies should consider the application of these methods, since they offer a powerful toolbox to analyze relational phenomena in the public health sector as well as in other bordering research areas as was demonstrated by this study.

## Supporting information

S1 Survey items Survey items for data collection. (PDF)

S1 Dataset Anonymized network matrix and organizational attributes. (XLSX)
