# Correction: Physical activity promotion in an urban district: Analyzing the mechanisms of interorganizational cooperation

**DOI:** 10.1371/journal.pone.0306487

**Published:** 2024-06-28

**Authors:** Hagen Wäsche, Laura Wolbring, Alexander Woll

In Table 3, the data in columns Estimate and SE of rows „Sector”heterophily and, Sports facility”activity are incorrect. Please see the correct Table 3 here.

**Table 3:** ERGM parameter estimates for the PAP network of cooperation.

	Model 1		Model 2	
Parameter	Estimate	*SE*	Estimate	*SE*
Cooperative ties (edges)	-5.34^*^	0.29	-5.90^*^	0.42
***Structural predictors***				
Centralization (preferential attachment)	0.71^*^	0.13	0.72^*^	0.14
Multiple triangulation (closure)	0.28	0.15	0.27^*^	0.13
***Attribute predictors***				
„Sector”heterophily			0.55^*^	0.26
„Sports facility”activity			0.15	0.13

*SE* = standard error,

^*^p < 0.05
