# Supplementary material for: Physical activity promotion in an urban district: Analyzing the mechanisms of interorganizational cooperation
Source: PLoS One. 2021 Nov 15;16(11):e0260053. doi: 10.1371/journal.pone.0260053 (PMC8592486; doi:10.1371/journal.pone.0260053)
Supplement: S1 Survey items — (PDF) [file pone.0260053.s001.pdf]

Physical activity promotion in an urban district: Analyzing the mechanisms of interorganizational cooperation.

Hagen Wäsche[1], Laura Wolbring[1], Alexander Woll[1]

[1]Institute of Sports and Sports Science, Karlsruhe Institute of Technology, Germany

Supporting information 1: Survey items for data collection

1. German version (original language)

1.1 Possession of sports facility

Verfügt Ihre Einrichtung über Sportstätten in der Oststadt?

Unter Sportstätten werden Sportanlagen (primär für den Sport geschaffen) oder Sportgelegenheiten (für andere Zwecke geschaffen, aber explizit dem Sport zur Verfügung stehende Räume, Plätze etc.) verstanden. Bitte geben Sie den Namen, Adresse, Art der Sportstätte sowie die jeweilige Größe in Quadratmetern (qm) an und ob sie für jedermann öffentlich zugänglich ist oder nicht. Berücksichtigen Sie dabei bitte, ob die Sportstätte im eigenen Besitz, zur Pacht oder in Pflege ist und machen Sie die Angaben im jeweiligen Feld.

	Name	Adresse	Art der Sportstätte	Größe in qm	Öffentlich zugänglich? (ja/nein)
im eigenen Besitz:					
im eigenen Besitz:					
im eigenen Besitz:					
zur Pacht/Miete:					
zur Pacht/Miete:					
zur Pacht/Miete:					
zur Pflege:					
zur Pflege:					
Zur Pflege:					

1.2 Identifying cooperation network

Mit welchen Einrichtungen kooperieren Sie im Rahmen Ihres Sportangebots in der Oststadt?

Bitte geben Sie an, mit welchen Einrichtungen (Schulen, Vereine, Kindergärten, freie Sportanbieter, Sportverwaltung, Kirchen und Altenpflegeeinrichtungen) Sie im Rahmen Ihres Sportangebots in der Oststadt kooperieren. Beziehen Sie sich dabei auf die Einrichtungen in nachfolgender Liste. Um die Liste zu betrachten, klicken Sie bitte auf den folgenden Hyperlink:

Liste der sportanbietenden und -verwaltenden Einrichtungen in oder mit Bezug zur Oststadt

Die Liste öffnet sich dann in einem neuen Fenster Ihres Browsers, während die Umfrage im Hintergrund geöffnet bleibt. Sie können bis zu zehn Einrichtungen nennen. Falls Sie mit mehr als zehn Einrichtungen kooperieren, nennen Sie nur die zehn wichtigsten. Beurteilen Sie für jede von Ihnen angeführte Einrichtung die Art der Kooperation (Mehrfachantworten sind möglich):

- es findet ein Austausch von Informationen statt

- es findet ein Austausch von Personal statt

- es findet eine Zusammenarbeit bei Sport- und Bewegungsangeboten statt
- es findet eine gemeinsame Nutzung von Sportstätten statt

	Austausch von Informationen	Austausch von Personal	Zusammenarbeit bei Angeboten	Nutzung von Sportstätten
Name der Einrichtung:	☐	☐	☐	☐
Name der Einrichtung:	☐	☐	☐	☐
Name der Einrichtung:	☐	☐	☐	☐
Name der Einrichtung:	☐	☐	☐	☐
Name der Einrichtung:	☐	☐	☐	☐
Name der Einrichtung:	☐	☐	☐	☐
Name der Einrichtung:	☐	☐	☐	☐
Name der Einrichtung:	☐	☐	☐	☐
Name der Einrichtung:	☐	☐	☐	☐
Name der Einrichtung:	☐	☐	☐	☐

2. English version

2.1 Possession of sports facility

Does your organization have sports facilities in the Oststadt?

Sports facilities are understood to be sports facilities (primarily created for sports) or sports opportunities (rooms, places, etc. created for other purposes, but explicitly available for sports). Please indicate the name, address, type of sports facility as well as the respective size in square meters (sqm) and whether it is open to the public or not. Please take into account whether the sports facility is owned, leased or maintained and provide the information in the respective field.

	name	address	type of sports facility	size in sqm	open to the public? (yes/no)
in own possession:					
in own possession:					
in own possession:					
leased:					
leased:					
leased:					
for maintenance:					
for maintenance:					
for maintenance:					

2.2 Identifying cooperation network

With which organizations do you cooperate as part of your sports offerings in the Oststadt?

Please indicate with which institutions (schools, sports clubs, kindergartens, commercial sports providers, sports administration, churches and old people’s homes) you cooperate as part of your

sports offering in the Oststadt. Refer to the organizations in the list below. To view the list, please click on the following hyperlink:

List of sports-providing and -administrating organizations in or related to the Oststadt.

The list will open in a new window in your browser while the survey remains open in the background. You can name up to ten organizations. If you cooperate with more than ten organizations, name only the most important ten. For each organization you name, rate the type of cooperation (multiple answers are possible):

- there is an exchange of information

- there is an exchange of personnel

- there is a cooperation in the provision of sports and physical activity programs

- there is a joint use of sports facilities

	exchange of information	exchange of personnel	cooperation on programs	use of sports facilities
name of the organization: [ ]	◻	◻	◻	◻
name of the organization: [ ]	◻	◻	◻	◻
name of the organization: [ ]	◻	◻	◻	◻
name of the organization: [ ]	◻	◻	◻	◻
name of the organization: [ ]	◻	◻	◻	◻
name of the organization: [ ]	◻	◻	◻	◻
name of the organization: [ ]	◻	◻	◻	◻
name of the organization: [ ]	◻	◻	◻	◻
name of the organization: [ ]	◻	◻	◻	◻
name of the organization: [ ]	◻	◻	◻	◻
